# Association between antihypertensive drugs and oral cancer: a drug target Mendelian randomization study

**DOI:** 10.3389/fphar.2023.1294297

**Published:** 2023-11-27

**Authors:** Junfeng Guo, Rongxing Liu, Fangfang Sheng, Qiuxiang Wu, Rufu Xu, Haitao He, Gang Zhang, Junjie Huang, Zhe Zhang, Rong Zhang

**Affiliations:** ^1^ Department of Pharmacy, Xinqiao Hospital, Third Military Medical University, Chongqing, China; ^2^ Department of Stomatology, The 970th Hospital of the Joint Logistics Support Force, Yantai, China; ^3^ Department of Stomatology, Xinqiao Hospital, Third Military Medical University, Chongqing, China

**Keywords:** antihypertensive drugs, blood pressure, oral cancer, Mendelian randomization, causal effect

## Abstract

**Background:** Recent reports have suggested that antihypertensive drugs may play an oncogenic role in common cancers, but it is still uncertain whether this could influence the risk of oral cancer. Through two-sample Mendelian randomization (MR), we sought to assess the causal effect of antihypertensive drugs on oral cancer outcomes.

**Methods:** To proxy the exposure of antihypertensive drugs, we utilized two genetic instruments, including expression quantitative trait loci of drug target genes and genetic variants within or around drug target genes related to blood pressure from genome-wide association studies. Inverse-variance-weighted MR (IVW-MR) and summary-data-based MR (SMR) were employed to compute the instrument effect estimates.

**Results:** It was observed through IVW-MR analysis that there is a positive relationship between KCNH2 (target of beta-adrenoceptor blockers)–mediated blood pressure and oral cancer (odds ratio [OR] = 1.197, 95% confidence interval [CI] = 1.028–1.394). Similarly, SMR analysis demonstrated that a higher expression of KCNH2 (target of beta-adrenoceptor blockers) was linked to a greater risk of oral cancer (OR = 2.223, 95% CI = 1.094–4.516). Both analyses yielded no consistent evidence of other associations.

**Conclusion:** This two-sample MR study proposed a latent causal association between KCNH2 (target of beta-adrenoceptor blockers) inhibition and diminished risk of oral cancer.

## 1 Introduction

Data from GLOBOCAN 2020 suggests that oral cancer is the 16th most common type of malignancy worldwide, with an estimated 377,713 new cases annually (Sung et al., 2021; Warnakulasuriya and Kerr, 2021). Oral cancer is becoming a major public health problem, particularly among young men and women, with an increasing prevalence (Sarode et al., 2020). It is a malignancy of epithelial origin, caused by a variety of factors, including genetics, epigenetics, habitual use of tobacco, areca nut, alcohol, microbial agents, and metabolic disorders such as hypertension, hyperglycemia, and dyslipidemia (Verhulst et al., 2019; Seo et al., 2020; Kumari et al., 2022).

Hypertension is a major contributor to cardiovascular complications, accounting for over 10.8 million deaths annually worldwide (GBD, 2019 Risk Factors Collaborators, 2020; Roth et al., 2020). It is a global health problem, affecting 31.1% of adults, and is marked by a high incidence, disability and mortality rate, and low awareness rate (Williams et al., 2018; Wu et al., 2018; Mills et al., 2020). A nationwide population-based study found a significant linear correlation between a 10 mmHg increase in diastolic blood pressure (DBP) and oral cancer in hypertensive patients (Seo et al., 2020). This emphasizes the need to actively control blood pressure to prevent oral cancer. Currently, clinical trials of antihypertensive drugs have been conducted to determine their efficacy and safety; however, there is an increasing focus on the potentially harmful effects these drugs may have on certain types of cancers. A meta-analysis demonstrated that a long-term intake of antihypertensive drugs can increase the risk of kidney cancer (Xie et al., 2020). In addition, antihypertensive drugs may also increase the risk of prostate cancer (Cao et al., 2018). However, there is no evidence of a correlation between oral cancer and antihypertensive drugs. Previous systematic reviews and meta-analyses of antihypertensive drugs have shown that obtaining reliable evidence regarding the risk of neoplasms is complex. Moreover, traditional drug epidemiology research is open to various biases, including immortal time bias, selection bias, and residual or unmeasured confounding, which may influence the precision and trustworthiness of the results.

Mendelian randomization (MR) is a measure for analyzing the causal effects of exposures on disease outcomes, which uses randomly assigned genetic variants inherited from parents as proxies for the exposures (Sanderson et al., 2022). Allocating genetic alleles randomly eliminates any influence of unknown confounding variables and minimizes the occurrence of measurement errors, as is the case with randomization in randomized controlled trials (RCTs) (Walker et al., 2017). Genetic variants of antihypertensive drug targets can be used as proxies to study the impact of their therapeutic inhibition on disease outcomes (Yarmolinsky et al., 2018). Drug target MR can be employed to anticipate drug development and repurposing prospects by utilizing genetic instruments near or within the target genes to simulate the potential effects of drug targets (Ference et al., 2016).

Our study used two-sample MR to analyze the association between antihypertensive drugs and oral cancer to provide useful guidance for the use of antihypertensive drugs. Furthermore, these findings may promote the reutilization of antihypertensive agents as potential oral cancer prevention strategies for future trial designs.

## 2 Materials and methods

### 2.1 Study design

**FIGURE 1:**
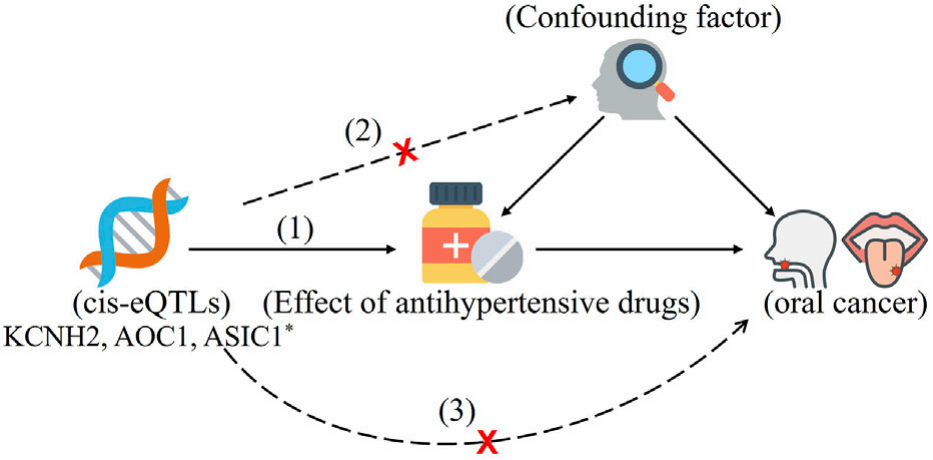
The drug-target Mendelian randomization framework in this study. Three assumptions are often required to make causal inference: (1) The chosen instrument is forecastable of the exposure. (2) The instrument is independent of confounding factors. (3) There is no horizontal pleiotropy (the instrument and the outcome are related solely through the exposure). *Common eQTLs SNPs significantly associated with the expression of KCNH2, AOC1, and ASIC1 in blood. They are targets corresponding to antihypertensive drugs.

Our two-sample MR study was based on expression quantitative trait loci (eQTLs) studies and summary-level data from genome-wide association studies (GWAS), and this data are publicly available (Figure 1; Supplementary Table S1). All these studies had the necessary approval from the relevant institutional review boards, and the participants had given their informed consents.

### 2.2 Selection of genetic instruments

This study included three common types of antihypertensive drugs: beta-adrenoceptor blockers, potassium-sparing diuretics, and sodium-channel blockers. The publicly accessible eQTL data and DrugBank database (https://www.drugbank.ca/) were used to identify target genes of the active ingredients in each of the antihypertensive drugs (Table 1). We employed available eQTLs for drug target genes as a proxy of exposure to antihypertensive drugs, and obtained the eQTLs summary-level data from the eQTLGen Consortium (https://www.eqtlgen.org/) (Supplementary Table S1). We identified common eQTLs single-nucleotide polymorphisms (SNPs) significantly associated with the expression of KCNH2, AOC1, and ASIC1 in blood (minor allele frequency [MAF] > 1%, *p* < 5 × 10^−8^) (Table 2). For this study, only cis-eQTLs, which were eQTLs located within 1 Mb on either side of the encoded gene, were incorporated to generate genetic instruments.

**TABLE 1:** Drug categories and target genes with their DrugBank ID.

Drug categories	One of target gene	Actions
Beta-adrenoceptor blockers	KCNH2	Inhibitor
Potassium-sparing diuretics	AOC1	Inhibitor
Sodium-channel blockers	ASIC1	Inhibitor

**TABLE 2:** Details of genetic instruments.

Exposure	Genetic instruments	
	Genetic variants associated with mRNA expression levels (cis-eQTLs)	Genetic variants associated with diastolic blood pressure (DBP)
Beta-adrenoceptor blockers	337 common cis-eQTLs (MAF >1%) in blood for KCNH2 gene (*p* < 5 × 10^−8^), top SNP: rs4725984	18 common SNPs (MAF >1%) in low linkage disequilibrium (*r* ^2^ < 0.3), associated with DBP (*p* < 5 × 10^−8^), located within ±100 kb windows from KCNH2 region
Potassium-sparing diuretics	1430 common cis-eQTLs (MAF >1%) in blood for AOC1 gene (*p* < 5 × 10^−8^), top SNP: rs7806458	11 common SNPs (MAF >1%) in low linkage disequilibrium (*r* ^2^ < 0.3), associated with DBP (*p* < 5 × 10^−8^), located within ±100 kb windows from AOC1 region
Sodium-channel blockers	179 common cis-eQTLs (MAF >1%) in blood for ASIC1 gene (*p* < 5 × 10^−8^), top SNP: rs590460	6 common SNPs (MAF >1%) in low linkage disequilibrium (*r* ^2^ < 0.3), associated with DBP (*p* < 5 × 10^−8^), located within ±100 kb windows from ASIC1 region
Statistical analyses		
Primary analysis	SMR	IVW MR
Sensitivity analyses	F-Statistic	F-Statistic
	Positive control analysis: DBP used as outcome	Positive control analysis: CHD used as outcome
	Linkage disequilibrium test: HEIDI test	Heterogeneity test: Cochran Q test
		Horizontal pleiotropy test: MR-Egger regression, MR-PRESSO test

CHD, coronary heart disease; DBP, diastolic blood pressure; eQTLs, expression quantitative trait loci; HEIDI, heterogeneity in dependent instruments; IVW-MR, inverse-variance-weighted Mendelian randomization; MAF, minor allele frequency; MR-PRESSO, Mendelian Randomization Pleiotropy RESidual Sum and Outlier; SMR, summary-data-based Mendelian randomization; SNPs, single-nucleotide polymorphisms.

As shown in Table 2, to confirm the detected connection utilizing the eQTLs as an instrument, we proposed an instrument to proxy the exposure of antihypertensive drugs by selecting SNPs associated with DBP at the genome-wide significance level (*p* < 5 × 10^−8^) within a 100 kb window of the target gene for each drug. A sample size of 757,601 from the International Consortium of Blood Pressure’s GWAS summary data for DBP was utilized to identify these SNPs, including only common SNPs (MAF >1%) (Evangelou et al., 2018). We permitted the SNPs used as instruments to be in low weak linkage disequilibrium (*r* ^2^ < 0.3) with each other, to obtain the greatest effectiveness of the instrument for each drug.

### 2.3 Outcome sources

The GWAS summary-level data for oral cancer originated from the NHGRI-EBI Catalog of published GWAS (GWAS Catalog), which includes 463,963 Europeans from Croatia, the Czech Republic, France, Germany, Greece, Italy, the Netherlands, Norway, Poland, the Republic of Ireland, Romania, the Russian Federation, Slovakia, Spain, Sweden, Canada, and the United Kingdom. To reduce the risk of collider bias and enable population-level comparisons, we included individuals without oral cancer as controls for all outcomes. All GWAS summary data sources are detailed in Supplementary Table S1.

### 2.4 Statistical analyses

#### 2.4.1 Primary analysis

First, employing eQTL as an instrument, a summary-data-based MR (SMR) method was utilized to generate effect estimates. In addition, this method utilizes summary-level data from GWAS and eQTL studies to analyze the connection between gene expression levels and the outcomes of interest (Zhu et al., 2016). SMR software (version 1.03) was employed for allele harmonization and analysis. Second, employing genetic variants associated with DBP as an instrument, an inverse-variance-weighted MR (IVW-MR) method was utilized to combine effect estimates (Burgess et al., 2013). The TwoSampleMR package (version 0.5.7) in R software was employed for allele harmonization and analysis.

#### 2.4.2 Sensitivity analyses

To minimize weak instrument bias, SNPs with an F-statistic >10 were included, and the F-statistic was used to assess the strength of the SNPs employed as the instrument (Burgess et al., 2011). We validated the accuracy of both genetic instruments through positive control analyses. Due to the definite antihypertensive effect of antihypertensive drugs, as a positive control study for the instrument from eQTLs, we examined the relationship between the exposure of interest and DBP. Due to the fact that coronary heart disease (CHD) is one of the main indications for antihypertensive drugs, we examined the relationship between the exposure of interest and CHD as a positive control study for the instruments from the DBP GWAS (Ettehad et al., 2016; Dézsi and Szentes, 2017). The GWAS summary data for CHD from the Coronary ARtery DIsease Genome wide Replication and Meta-analysis plus The Coronary Artery Disease Genetics (CARDIoGRAMplusC4D) Consortium, including 184,305 samples (Nikpay et al., 2015).

In the SMR method, we used the heterogeneity in dependent instruments (HEIDI) test to examine whether the association between gene expression and results was caused by linkage scenarios. A *p*-value of more than 0.05 in the HEIDI test suggests that the association is not due to linkage. In the IVW-MR method, the Cochran Q test is used to determine the presence or absence of heterogeneity, with *p* > 0.05 indicating no heterogeneity. We used MR-PRESSO analysis and MR-Egger regression to evaluate the potential horizontal pleiotropy of the SNPs used as instrument variants. MR-PRESSO analysis with a global test of *p* < 0.05 indicates the existence of horizontal pleiotropic outliers, while MR-Egger regression with *p* < 0.05 suggests horizontal pleiotropic validity.

Taking multiple testing into account, Bonferroni correction was employed to modify the thresholds of significance level, thus providing a strong evidence for *p* < 0.017 (3 exposures and 1 outcome) and a suggestive evidence for 0.017 ≤ *p* < 0.05.

## 3 Results

### 3.1 Genetic instruments selection and oral cancer

We identified 337, 1430, and 179 cis-eQTLs of drug target gene KCNH2, AOC1, and ASIC1 from the eQTLGen Consortium, and selected the most significant cis-eQTL SNP as a genetic instrument for each drug target gene (Table 2; Supplementary Table S2). In addition, we selected 18, 11, and 6 SNPs within or nearby gene KCNH2, AOC1, and ASIC1 from a GWAS summary data of DBP in the International Consortium of Blood Pressure, respectively (Table 2; Supplementary Table S3). Our F-statistics for all instrument variants were greater than 10, indicating that our study can effectively reduce weak instrument bias (Supplementary Tables S2, S3). Results from the positive control study indicated a significant correlation between exposure to each drug and DBP when eQTLs-proposed instruments were employed (Supplementary Table S4), as well as between exposure to each drug and CHD when DBP GWAS-proposed instruments were employed (Supplementary Table S5). This further confirmed the potency of the chosen genetic instruments.

### 3.2 Primary analysis

**FIGURE 2:**
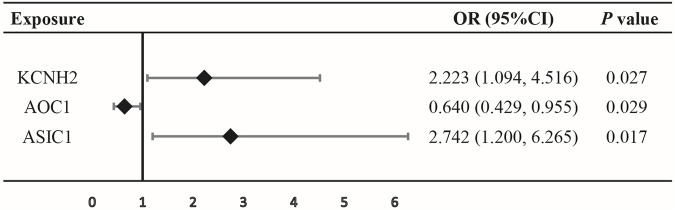
Association between expression of gene KCNH2, AOC1, or ASIC1 and oral cancer outcomes by summary-data-based Mendelian randomization (SMR).

SMR analysis observed a suggestive evidence that the increased expression of the KCNH2 and ASIC1 genes in blood is linked to an increased risk of oral cancer (KCNH2: OR = 2.223, 95% CI = 1.094–4.516, *p* = 0.027; ASIC1: OR = 2.742, 95% CI = 1.200–6.265, *p* = 0.017), while lower expression of the AOC1 gene was associated with a decreased risk of oral cancer (OR = 0.640, 95% CI = 0.429–0.955, *p* = 0.029) (Figure 2; Supplementary Table S2), indicating that inhibition of KCNH2 and ASIC1 might lower the risk of oral cancer, while upregulation of AOC1 could increase the risk of cancer.

**FIGURE 3:**
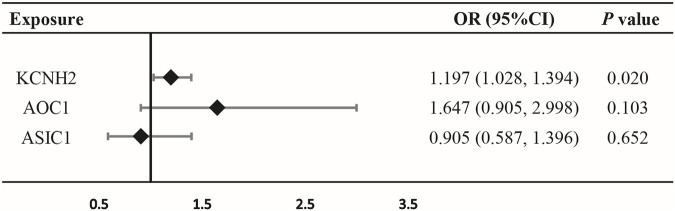
Association between diastolic blood pressure (DBP) mediated by gene KCNH2, AOC1, or ASIC1 and oral cancer outcomes by inverse-variance-weighted Mendelian randomization (IVW-MR).

IVW-MR analysis also provided suggestive evidence of an association between KCNH2-mediated DBP and risk of oral cancer (OR = 1.197, 95% CI = 1.028–1.394, *p* = 0.020) (Figure 3; Supplementary Table S6), suggesting that KCNH2 inhibition may be a protective factor against oral cancer. However, no evidence was found, by IVW-MR analysis, to suggest a connection between AOC1, ASIC1-mediated DBP and oral cancer (Figure 3; Supplementary Table S6).

### 3.3 Sensitivity analysis

In the SMR method, the HEIDI test indicated that none of the associations observed were caused by a linkage (*p* > 0.05, Supplementary Table S2).

In the IVW-MR method, Cochran Q test did not reveal any heterogeneity among the reported results (KCNH2, *p* > 0.05, Supplementary Table S6). MR-PRESSO analysis and MR-Egger regression both indicated that there was no significant overall horizontal pleiotropy in the intercept term (*p* > 0.05, Supplementary Table S6).

## 4 Discussion

We employed GWAS summary data and cis-eQTL in a two-sample MR analysis to extrapolate the potential impact of antihypertensive drugs on oral cancer. Our study provides suggestive evidence that KCNH2 expression and KCNH2-mediated DBP are positively correlated with oral cancer risk, both of which collectively indicate the potential protective impact of KCNH2 inhibition on oral cancer (OR _instrument 1_ = 0.450, 95% CI = 0.221–0.914; OR _instrument 2_ = 0.835, 95% CI = 0.717–0.973). We also observed suggestive evidence of a positive relationship between ASIC1 expression and oral cancer, although this was not validated when using DBP GWAS as an instrument. On the other hand, suggestive evidence that AOC1 expression has a negative association with oral cancer was found; however, this association was not corroborated when using DBP GWAS as an instrument.

Beta-adrenoceptor blockers have been around for years and remain one of the most commonly used medications for cardiovascular diseases. It is recommended as a first-line treatment for hypertension (Chrysant and Chrysant, 2012). Studies have shown that beta-adrenoceptor blockers act on KCNH2, leading to a reduction in blood pressure due to the inhibition of KCNH2 (Bodi et al., 2013). In addition, we also confirmed that KCNH2 is the target gene for beta-adrenoceptor blockers in the DrugBank database. Therefore, this MR study utilized genetic variants linked to KCNH2 expression or KCNH2-mediated DBP as instruments to proxy the exposure of antihypertensive drugs (beta-adrenoceptor blockers). Suggestive evidence from both analyses suggests that KCNH2 inhibition may lower the risk of oral cancer.

The potential association between antihypertensive drugs and cancer risk has been a source of great concern (Tadic et al., 2019; Heisel et al., 2023; Lin et al., 2023; Wang et al., 2023). The results of prior observational studies on the carcinogenic risk of beta-adrenoceptor blockers have been somewhat inconsistent. It has been reported that the use of beta-adrenoceptor blockers could be correlated with a greater risk of breast cancer (Zheng et al., 2021). However, beta-adrenoceptor blockers have also been thought of as potential new treatments for cancer (Engineer et al., 2013). Moreover, a prospective cohort study of 839 individuals monitored over the course of 10 years revealed that beta-adrenoceptor blockers may lead to a decreased risk of cancer (Algazi et al., 2004). Despite the absence of strong evidence, our results provide causal evidence supporting the findings from the above cohort study.

Numerous investigations have assessed the association between antihypertensive drugs and cancer, comprising of randomized controlled studies, basic research, and epidemiological data (Kidoguchi et al., 2022). However, there is currently no relevant study on the relationship between antihypertensive drugs and risk of oral cancer. Our study demonstrated the potential causal relationship between antihypertensive drugs and a decreased risk of oral cancer by using two-sample MR. This is the first and innovative endeavor to investigate the effect of antihypertensive drugs on oral cancer. We employed genetic instruments to proxy drug exposure, aiming to preclude any reverse causal relationships and to minimize confounding bias. In addition, two different genetic instruments were utilized to proxy the studied drugs, which helped to validate each other’s effectiveness estimates. Moreover, sensitivity analyses were conducted to assess the potency of the genetic instruments and the hypothesis of the MR studies.

Despite its novelty, there are some limitations that should be considered when interpreting the results of our study. First, drug-target MR analysis may overestimate the effect of short-term medication use, as they reflect the cumulative, long-term effect of drug target alteration (Zheng et al., 2017). As a result, this study could be more effective in suggesting directions for causal connections of the drugs. Second, there are various classes of antihypertensive drugs, and our study only found a relationship between beta-adrenoceptor blockers and the risk of oral cancer. However, different beta-adrenoceptor blockers have varied pharmacological and pharmacokinetic properties (Khouri et al., 2016). These differing pharmacological properties (e.g., differential absorption rate and plasma half-life) can influence the therapeutic benefit (or experience of adverse effects) of beta-adrenoceptor blockers (Lin et al., 2017). Future evaluation of the potential effects of long-term use of beta-adrenoceptor blockers on cancer risk should therefore include assessment of whether findings are specific to individual agents or classes of beta-adrenoceptor blockers. Third, the efficacy of antihypertensive drugs (beta-adrenoceptor blockers) may differ among subgroups. Nevertheless, since we used data at a summary level, we were not able to carry out subgroup analyses. Consequently, individual level data are needed to gain a more comprehensive understanding in further MR studies. Fourth, despite the multiple sensitivity analyses we conducted to assess the assumptions of the MR study, it was not possible to completely exclude horizontal pleiotropy and/or confounding bias. Fifth, caution should be taken when attempting to apply these findings to other populations, because the study was based primarily on eQTLs and GWAS data from a European population. Sixth, there remains a risk of false-positive findings when examining the protective effect of antihypertensive drugs (beta-adrenoceptor blockers) on oral cancer by applying the Bonferroni correction for multiple tests. However, these findings are preliminary and require confirmatory evidence (from long-term follow-up of clinical trials) in order to guide clinical decision-making.

## 5 Conclusion

Our findings present convincing evidence that beta-adrenoceptor blockers are associated with a decreased risk of oral cancer in the European population. In addition, it offers a bright therapeutic prospect for the prevention of oral cancer. Further studies should be conducted to investigate the potential of retargeting or repurposing antihypertensive drugs to expedite the drug development process for oral cancer.

## Data Availability

Publicly available datasets were analyzed in this study. This data can be found here: https://www.eqtlgen.org/cis-eqtls.html, http://ftp.ebi.ac.uk/pub/databases/gwas/summary_statistics/, https://www.ukbiobank.ac.uk/, and http://www.cardiogramplusc4d.org/.

## Abbreviations

CI, confidence interval; CHD, coronary heart disease; DBP, diastolic blood pressure; eQTLs, expression quantitative trait loci; GWAS, genome-wide association studies; HEIDI, heterogeneity in dependent instruments; IVW-MR, Inverse-variance-weighted Mendelian randomization; MAF, minor allele frequency; MR, Mendelian randomization; MR-PRESSO, Mendelian Randomization Pleiotropy RESidual Sum and Outlier; OR, odds ratio; RCTs, randomized controlled trials; SMR, summary-data-based MR; SNPs, single-nucleotide polymorphisms.
